# Potential Role of Methylation Marker in Glioma Supporting Clinical Decisions

**DOI:** 10.3390/ijms17111876

**Published:** 2016-11-10

**Authors:** Krzysztof Roszkowski, Jacek Furtak, Bogdan Zurawski, Tadeusz Szylberg, Marzena A. Lewandowska

**Affiliations:** 1 Department of Oncology, Radiotherapy and Ginecologic Oncology, Faculty of Health Sciences, Nicolaus Copernicus University, Collegium Medicum, 85-796 Bydgoszcz, Poland; roszkowskik@cm.umk.pl; 2 Department of Neurosurgery, Military Clinical Hospital, 85-681 Bydgoszcz, Poland; jacekf67@poczta.onet.pl; 3 Outpatient Chemiotherapy The F. Lukaszczyk Oncology Center, 85-796 Bydgoszcz, Poland; bzur@wp.pl; 4 Department of Pathomorphology, Military Clinical Hospital, 85-681 Bydgoszcz, Poland; szylberg@10wsk.mil.pl; 5 Molecular Oncology and Genetics Department, The F. Lukaszczyk Oncology Center, 85-796 Bydgoszcz, Poland; 6 Department of Thoracic Surgery and Tumors, Faculty of Medicine, Nicolaus Copernicus University, Collegium Medicum, 85-796 Bydgoszcz, Poland

**Keywords:** glioma, *IDH1*, *MGMT*, radiotherapy, survival

## Abstract

The *IDH1*/2 gene mutations, *ATRX* loss/mutation, 1p/19q status, and *MGMT* promoter methylation are increasingly used as prognostic or predictive biomarkers of gliomas. However, the effect of their combination on radiation therapy outcome is discussable. Previously, we demonstrated that the *IDH1* c.G395A; p.R132H mutation was associated with longer survival in grade II astrocytoma and GBM (Glioblastoma). Here we analyzed the *MGMT* promoter methylation status in patients with a known mutation status in codon 132 of *IDH1*, followed by clinical and genetic data analysis based on the two statuses. After a subtotal tumor resection, the patients were treated using IMRT (Intensity-Modulated Radiation Therapy) with 6 MeV photons. The total dose was: 54 Gy for astrocytoma II, 60 Gy for astrocytoma III, 60 Gy for glioblastoma, 2 Gy per day, with 24 h intervals, five days per week. The patients with *MGMT* promoter methylation and *IDH1* somatic mutation (OS = 40 months) had a better prognosis than those with *MGMT* methylation alone (OS = 18 months). In patients with astrocytoma anaplasticum (*n* = 7) with the *IDH1* p.R132H mutation and hypermethylated *MGMT*, the prognosis was particularly favorable (median OS = 47 months). In patients with astrocytoma II meeting the above criteria, the prognosis was also better than in those not meeting those criteria. The *IDH1* mutation appears more relevant for the prognosis than *MGMT* methylation. The *IDH1* p.R132H mutation combined with *MGMT* hypermethylation seems to be the most advantageous for treatment success. Patients not meeting those criteria may require more aggressive treatments.

## 1. Introduction

DNA methylation plays a crucial role in many biological processes—this epigenetic modification contributes in the temporal and spatial regulation of gene expression [1], X chromosome inactivation [2], genome imprinting [3], male infertility [4] and carcinogenesis [5], as reviewed in [6]. It has been observed that cancer cells have an excessive methylation of genes involved in cell cycle regulation (*p16INK4a*, *p15INK4a*, *Rb*, *p14ARF*), DNA damage repair (*BRCA1*, *MGMT*), apoptosis (*DAPK*, *TMS1*), detoxification, cell differentiation, angiogenesis and metastasis [7]. The CpG islands hypermethylation of the promoter region of tumor suppressor genes is a key mechanism of gene activation in cancer [8,9]. On the other hand, hypomethylation is a player in oncogenesis through the activation of oncogenes, such as *cMYC* and *H-RAS* [10], as well as chromosomal instability [11].

Glioblastoma multiforme was the first cancer selected for epigenome analysis in the pilot The Cancer Genome Atlas (TCGA) project. Analysis of the promoter DNA methylation alteration in 272 glioblastoma tumors identified a group of patients with a repetitive methylation pattern called glioma-CpG island methylator phenotype (G-CIMP phenotype). These G-CIMP tumors had distinct molecular features, including a high frequency of mutations in the *IDH1* gene and characteristic copy number alterations. Patients with the G-CIMP phenotype were younger at the time of diagnosis in a statistically significant manner and presented significantly improved outcomes [5]. Our previous observation in a Polish group of glioma patients was consistent with those results. The *IDH1* c.G395A; p.R132H mutation was associated with longer survival in grade II astrocytoma, glioblastoma, and pooled groups of patients with WHO grade II glioma [12]. Characterization of the molecular landscape of lower-grade gliomas confirmed the presence of a known *IDH1* mutation and 1p/19q codeletion (whole-arm loss of the long arm of chromosome 1 and the short arm of chromosome 19) and their role as a predictive markers [13,14]. The latest evaluation of molecular subgroups of gliomas identified *ATRX*, *CIC*, and *FUBP1* mutations, and allowed distinguishing patients with *IDH1*/*CIC*/*FUBP1* or *IDH1*/*ATRX* mutations [15]. The first group had the longest median overall survival (96 months) in comparison with patients with *IDH1* mutations and *ATRX* loss (51 months), while the shortest median overall survival was in glioma patients who did not harbor any of those signatures (13 months) [15].

In the presented study, we analyzed the *MGMT* methylation levels in 83 glioma patients with a known *IDH1* mutation status and re-evaluated the clinical data with two years extended novel observations.

## 2. Results

### 2.1. Patient Characteristics

In this study, based on our previously reported group of 139 glioma patients [12], we randomly selected 90 patients for further evaluation of *MGMT* promoter methylation. Seven patients were excluded and 83 patients were screened for *MGMT* methylation. Additional clinical features of the patients with *MGMT* methylation and *IDH1* statuses, including a small number of patients treated with temozolomide, are shown in Table 1. Among the chosen 83 patients, 55 had a previously detected *IDH1* c.G395A; p.R132H mutation and 28 were wild-type in codon 132 of the *IDH1* gene [12]. Among the histological types, we evaluated *MGMT* promoter methylation in 57 astrocytomas WHO II, 13 astrocytomas WHO III and 13 glioblastomas (WHO IV). The frequency of *MGMT* hypermethylation was observed in 53% astrocytoma III and GBM samples, and the highest hypermethylation was observed in astrocytoma II (61.4%) (Table 2).

### 2.2. Molecular Evaluation

We previously demonstrated that the *IDH1* c.G395A; p.R132H mutation is more frequently observed in higher-grade astrocytoma cases. In the subsequent studies, we showed that methylation correlates with the presence of the somatic mutation in *IDH1*:37/55 samples (67.27%), with the somatic mutation having the *MGMT* promoter methylated. On the other hand, 12/28 (42.85%) samples confirmed as wild-type *IDH1* had their *MGMT* promoter methylated. Moreover, we evaluated the methylation status based on the comparison between the methylated (M) and unmethylated (U) amplicons for each of the 83 randomly selected DNA samples. With the M/U ratio of >1, the *MGMT* promoter methylation status was evaluated as high. Such a high methylation was observed in 53.6% of *IDH1* pR132H samples (30/56 total) and in 18.5% (5/27) of Wild Type *IDH1* samples. On the contrary, low methylation status (M/U < 1) was found mainly in samples with WT *IDH1* (55.5%; 15/27) and was definitely less frequent (34%; 19/56) in tumor samples with the somatic mutation in the *IDH1* codon 132 (Table 3).

### 2.3. Overall Survival

**Figure 1:**
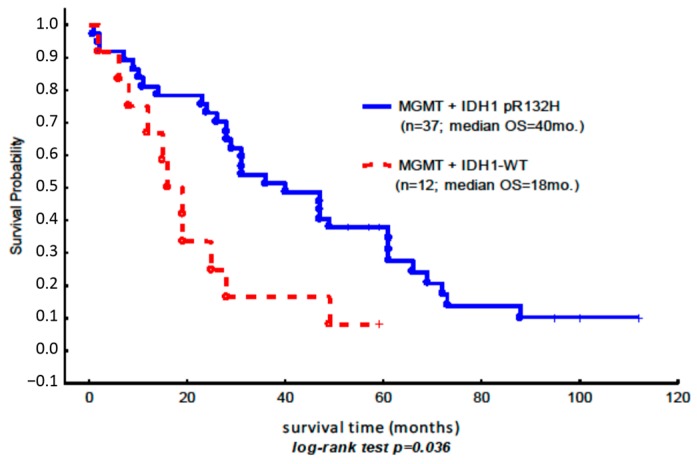
Kaplan–Meier overall survival curves for the subgroup of patients with *MGMT* promoter methylation, in which the assessment of the methylation status based on the comparison between the methylated (M) and unmethylated (U) amplicons was equal to or higher than 1 (Table 3). The blue line shows the Kaplan–Meier overall survival curves for the subgroup of patients with the somatic mutation *IDH1* p.R132H and the concurrent hypermethylation of *MGMT* (*n* = 37 (7 + 30)); the red line indicates the methylation of *MGMT* (*n* = 12 (7 + 5)) and the wild-type *IDH1* gene (Table 3).

In the studied population, the patients with methylation in the *MGMT* gene promoter and the concurrent somatic mutation in the *IDH1* gene (OS = 40 months) had a better prognosis than the subgroup of patients with the methylated *MGMT* gene alone (OS = 18 months) (Figure 1).

In a small subgroup of patients with astrocytoma anaplasticum (*n* = 7) characterized by the presence of the somatic mutation *IDH1* p.R132H and the concurrent intermediate degree of *MGMT* methylation, the prognosis was particularly favorable (median OS = 47 months) compared to the other subgroups of patients (Table 2).

## 3. Discussion

The *IDH1*/2 mutations, 1p/19q status and *MGMT* promoter methylation are commonly recognized biomarkers in patients with gliomas [16,17,18]. Their role as prognostic or predictive biomarkers—that can be used in diagnostics—is growing. However, the question of how the outcome of radiation therapy depends on the combination of these two biomarkers remains open and is still debatable. It was demonstrated that in malignant gliomas of WHO grades III/IV with the *IDH1* mutation, *MGMT* promoter methylation was associated with prolonged Progression-free survival (PFS) with chemotherapy ± radiation therapy (RT) or RT-only groups [19]. Moreover, a recent investigation (in which a molecular registry of 274 glioblastoma patients was used) showed that the poorest OS and PFS were observed in wild-type *IDH1* glioblastomas with an unmethylated *MGMT* promoter [18]. Our results are in concordance with the above results: the best OS was observed in all patients, regardless of their histopathological type, who had hypermethylated *MGMT* and mutated *IDH1*, with a median of 33.5 months (*n* = 30). Furthermore, the status of the *IDH1* mutation is more relevant for the prognosis than the methylation of *MGMT*.

In the subgroup with astrocytoma II, patients meeting the above criteria also had a considerably better prognosis than those not meeting those criteria (Table 2). In every histological subgroup, e.g., that with astrocytoma II, patients with a better prognosis were those with the *IDH1* p.R132H mutation, regardless of the *MGMT* status, compared to those with wild-type *IDH1*. The most advantageous situation for RT treatment success seems to be the combination of the somatic mutation *IDH1* p.R132H with the hypermethylation of *MGMT*. In an important report by Yang et al., a role of the mutated *IDH1* gene in the resistance to temozolomide therapy is suggested. The article confirms the hypothesis in astrocytoma/glioblastoma cell lines, in which exogenous expression of the mutated *IDH1* results in a three- to 10-fold increase in temozolomide resistance after long-term passage [18]. Unfortunately, our group of seven patients who received Temozolomide (TMZ) was too small for similar conclusions or any statistical evaluation (only three patients were diagnosed with astrocytoma II and 4 with GBM, and received chemotherapy; all of them, excluding one, had the mutation in *IDH1* and the methylated *MGMT*; data not shown).

As clinical and molecular data were updated for 83 patients in 2015, we can discuss whether adding the *MGMT* status brings light to future clinical decisions. Previously, we demonstrated that the median overall survival observed in astrocytoma II patients, grouped on the basis of the presence of the *IDH1* mutation only, was 24 months longer [12]. Currently, with the additional two years of clinical observation and division of the patients into groups with mutated/WT *IDH1* and methylated/nonmethylated *MGMT* genes, we observed a 28-month-longer overall survival between the *IDH1*-mutated and *MGMT*-methylated group of 24 patients versus the *IDH1* wild-type and *MGMT*-methylated group of 11 patients (Table 2). Furthermore, there was a 14-month difference in the median OS in the *IDH1*-mutated subgroup of patients with methylated *MGMT* versus the nonmethylated subgroup. We must also point it that a surprising proportion of *IDH* mutant gliomas are unmethylated—this may reflect the relative insensitivity of MS-PCR (methylation-specific polymerase chain reaction) compared to other methods, for example DNA pyrosequencing, for MGMT promoter methylation [20].

On the other hand, looking at the methylation level in the *IDH1*-WT subgroup of patients, we did not notice any significant variance between patients with different methylation status. In view of the above results, the somatic mutation *IDH1* p.R132H and the concurrent methylation of the *MGMT* promoter has the most favorable prognosis.

In conclusion, we found a small population of patients with astrocytoma anaplasticum (WHO grade III), characterized by the presence of the somatic mutation *IDH1* p.R132H and the concurrent hypermethylation of *MGMT*, for whom the prognosis was particularly favorable (median OS = 47 months). In the subgroup with astrocytoma II, patients meeting the above criteria also had a considerably better prognosis than those not meeting those criteria. The results indicate that the status of the *IDH1* mutation is more relevant for the prognosis than the methylation of *MGMT*, and this is consistent within every histological subgroup. The most advantageous situation for treatment success seems to be the combination of the somatic mutation *IDH1* p.R132H with the hypermethylation of *MGMT*. In view of the above results, patients not meeting those criteria may have to be subjected to more aggressive oncological treatments [14].

## 4. Materials and Methods

### 4.1. Patient Specimens

We randomly selected 83 patients with glioma (WHO II, III or IV) from a previously reviewed cohort of 139 patients with histopathologically confirmed glioma diagnosed between 2000 and 2011 [12]. Additionally, clinicopathological evaluation was performed in 2015 in order to update the data for further statistical analysis. All selected patients had a previously evaluated mutation status in codon 132 of isocitrate dehydrogenase 1 gene, and a novel *MGMT* promoter methylation analysis was conducted. The patients were divided into groups for the correlation of clinical and genetic data based on the molecular indications: the presence or absence of mutations in the *IDH1* gene, as well as methylation status. The degree of tumor resection was assessed using an MRI examination conducted within 12 h after the surgery. In all patients participating in the study, a subtotal tumor resection was conducted. Subsequently, the patients were treated postoperatively using a routine IMRT radiation therapy with 6 MeV photons within four weeks after the procedure. The total dose was: 54 Gy for astrocytoma II, 60 Gy for astrocytoma III, 60 Gy for glioblastoma, divided into fractions of 2 Gy per day, with 24 h intervals between exposures, five days per week. The Planning Target Volume (PTV) and critical organs included all structures in accordance with the ICRU Report 62 [21].

The clinical characteristics of the patients are presented in Table 1.

### 4.2. Methylation Analysis

For methylation analysis, we used previously isolated DNA with a known status of *IDH1* mutation. Then 1 μg of DNA was used as a template for bisulfite DNA conversion according to the manufacturer instructions EpiTect Bisulfite Kit, QIAGEN (Hilden, Germany). Subsequently, MS-PCR was conducted with sets of primers specific for the methylated and unmethylated sequence of the *MGMT* promoter, according to the previously described methylation-specific polymerase-chain-reaction assay [22,23,24]. The PCR products were evaluated using horizontal electrophoresis (stained with ethidium bromide and visualized under ultraviolet illumination). During methylation analysis, the intensity of the bands present on the gel (93 bp for unmethylated and 81 bp for methylated *MGMT* promoter sequence) was assessed using a five-point scale (from 1 to 5). As a control, ready-made samples of methylated DNA (EpiTect Control DNA, human, cat. no. 59665) and non-methylated DNA (EpiTect Control DNA, human, cat. no. 59655) were used.

### 4.3. Statistical Analysis

For statistical analysis, the STATISTICA (version 12.0; lic. no: JGVP502E256520ARCN-4) computer software (StatSoft, Inc., Tulsa, OK, USA). The association between overall survival and the status of the *IDH1* (isocitrate dehydrogenase 1) c.G395A; p.R132H mutation and *MGMT* promoter methylation were estimated using the Kaplan–Meier method and assessed using the log-rank test. The results were considered as statistically significant at *p* < 0.05.

## Figures and Tables

**Table 1:** Clinical features of the study population of 83 glioma patients.

No. of Patients	All Patients	83
Age	Median	36
	≤40	51
	>40	32
Gender	Male	48
	Female	35
Histopathological diagnosis	Astrocytoma II	57
	Astrocytoma III	13
	Glioblastoma	13
Treatment	Radiotherapy	83
	Chemotherapy	7
ECOG performance status	I	56
	II	25
	III	2
	IV	0

ECOG, Eastern Cooperative Oncology Group.

**Table 2:** Distribution of the mutation in *IDH1* and *MGMT* promoter methylation among the patients according to their histopathological diagnosis (WHO II, III, IV). OS, Overall Survival.

	Astrocytoma II (*n* = 57) (Median OS, Month)		Astrocytoma III (*n* = 13) (Median OS, Month)		Glioblastoma (*n* = 13) (Median OS, Month)	
	*MGMT*−	*MGMT*+	*MGMT*−	*MGMT*+	*MGMT*−	*MGMT*+
*IDH1* pR132H	33 (*n* = 11)	47 (*n* = 24)	28 (*n* = 5)	47 (*n* = 7)	22 (*n* = 2)	22.5 (*n* = 6)
*IDH1* WT	21 (*n* = 11)	19 (*n* = 11)	14 (*n* = 1)	0	7.5 (*n* = 4)	2 (*n* = 1)

**Table 3:** Assessment of the methylation status based on the comparison between the methylated (M) and unmethylated (U) amplicons for each of the 83 randomly selected DNA samples.

Methylation Ratio	M/U < 1	M/U = 1	M/U > 1
*IDH1* pR132H (*n* = 56)	*n* = 19	*n* = 7	*n* = 30
*IDH1* WT (*n* = 27)	*n* = 15	*n* = 7	*n* = 5
